# Low-Intensity Repetitive Exercise Induced Rhabdomyolysis

**DOI:** 10.1155/2015/281540

**Published:** 2015-11-26

**Authors:** Mina Tran, Nicholas Hayden, Brandon Garcia, Veronica Tucci

**Affiliations:** ^1^Baylor College of Medicine, Section of Emergency Medicine, 1504 Taub Loop, Houston, TX 77030, USA; ^2^Baylor College of Medicine, Section of Emergency Medicine, Summer Medical and Research Training (SMART), 1504 Taub Loop, Houston, TX 77030, USA

## Abstract

Rhabdomyolysis is a rare condition caused by the proteins of damaged muscle cells entering the bloodstream and damaging the kidneys. Common symptoms of rhabdomyolysis are muscle pain and fatigue in conjunction with dark urine; kidney damage is a common symptom among these patients. We present a case of a 23-year-old woman who displayed myalgia in the upper extremities caused by low-intensity and high-repetition exercise. She was successfully diagnosed and treated for exertional rhabdomyolysis. This patient had no significant medical history that would induce this condition. We urge the emergency medical community to observe and monitor patients that complain of myalgia to ensure they are not suffering from rhabdomyolysis even in atypical cases.

## 1. Case Study

Three days prior to arrival in the emergency room, a fit 23-year-old female patient exerted herself through low-intensity, high-repetition bicep curls with 10–15 lb weights after a hiatus of several months. Patient reported the use of creatine supplements in conjunction with her workout. The following day she noticed a great deal of soreness with tension in her upper extremities, specifically her biceps. The patient reported the incapability to fully extend her arms and commented that her urine was very dark and “tea color[ed].” She denied the occurrence of dysuria, fevers, chills, chest pain, and abdominal pain. However, the patient did report mild paresthesia in her arms prior to admission to the hospital, with improvement before examination.

Upon immediate physical examination, the patient had elevated blood pressure (142/115). Her biceps were prominent and tense and had a slight loss in sensation and her urine matched the description of being “tea color[ed].” The information from the patient history and physical examination heavily suggested that the patient suffered from rhabdomyolysis.

Following the working diagnosis, the laboratory reported that the patient's creatine kinase levels within the blood were 156,339 U/L and urinary analysis showed 3+ blood and 1 RBC. The presence of blood without RBC in the urine is consistent with a diagnosis of rhabdomyolysis. The patient began treatment for rhabdomyolysis with aggressive fluid resuscitation and strict urine ins and outs monitoring. These interventions lead to a downward trend of her creatine kinase levels to 68,4281 U/L. Through aggressive fluid resuscitation, the patient's creatine kinase levels plateaued with the closest to discharge creatine kinase level being 16,245 U/L. The patient was discharged 4 days after admission. Throughout her stay at the hospital, she was hemodynamically stable, despite initial admission to the ICU.

Using the correlation of the laboratory findings, the patient's history, and physical examination, we have evidence to believe that we were successful in identifying and treating a severe case of exertional rhabdomyolysis acquired through low-intensity and high-repetition exercise [1].

## 2. Discussion

Rhabdomyolysis is a disease caused by the rapid breakdown of muscle tissue released into the bloodstream. Myoglobin, a component protein of skeletal muscle tissue, in large amounts, can cause renal failure by accumulating in the kidneys and preventing the proper function of that organ [2]. The destruction of muscle tissue can be caused by a myriad of activities such as intense muscle strain, crush trauma, electrocution, and drug use [3]. Patients who develop rhabdomyolysis often feel extreme pain in the area of muscle death and display dark and concentrated urine, as well as nausea, difficulty concentrating, and weakness or fatigue. If treated quickly the treatment outcome is positive.

Rhabdomyolysis was first associated with renal failure in a report written by Bywaters and Beall during the Battle of Britain in 1940 [4]. They noticed that patients crushed under destroyed infrastructure suffered renal failure even in cases where the patients' kidneys were not harmed. Upon investigation Bywaters and Beall discovered that myoglobin, an iron and oxygen binding protein found in muscle fibers, had accumulated in the blood to levels higher than could be properly filtered by the kidneys [4]. The myoglobin then began to collect resulting in acute renal failure.

The disease is rather rare in the general population, appearing in about 0.0001% (25/25 million) of patients between 1990 and 1999 [5]. Early study of rhabdomyolysis was made possible by investigating the victims of natural disaster and trauma, but many studies have been completed demonstrating that extreme muscle strain can also cause the disease. In a 2015 retrospective study from 2007 to 2013, Dr. Imstepf et al. reviewed emergency department admissions after workouts and examined patients incidents. They found that 45% of the patients admitted were from 17 to 30 years of age, and the amount of rhabdomyolysis was a small portion of the total patients admitted. In conjunction with the recent data, this case study suggests that young patients suffer a significant portion of injury from working out and that, within the population, patients suffering from rhabdomyolysis are present [6].

Athletes, both professional and amateur, are susceptible to rhabdomyolysis after participating in extreme workout routines. The excessive breakdown of muscle caused by intense weight lifting releases myoglobin into the bloodstream and can lead to accumulation in the kidneys [7]. This risk is currently accepted within the medical community for patients who are hardcore athletes that lift large amounts of weight. However, the case we presented demonstrates that severe muscle strain does not have to be caused by heavy weights to develop into rhabdomyolysis; lifting light weight with many repetitions can cause enough muscle breakdown to induce rhabdomyolysis as well.

Low-weight, high-repetition workouts can cause just as much strain on the muscle fibers as high-weight workouts. The main cause of rhabdomyolysis, the extreme breakdown of muscle fibers, and release of myoglobin into the bloodstream in fact can be caused by many factors [3]. Physicians should concern themselves with looking at symptoms and should not discount the potential diagnosis of rhabdomyolysis when an athlete admits to the use of low amounts of weight in their workout routine or denies the use of heavy weights. ER doctors should add the consideration of rhabdomyolysis to their protocols whenever a patient presents with myalgia, fatigue, weakness, and other symptoms associated with this disease.

As described with the 23-year-old female patient, myalgia caused from low-intensity workouts can lead to rhabdomyolysis. When a patient presents with severe myalgia in the extremities, lab tests should be ordered to check for creatine levels in the blood to determine if the patient may have rhabdomyolysis [3]. Patients who meet the criteria for possible rhabdomyolysis should be given intravenous fluids and be monitored for 24 to 48 hours [7].

## 3. Conclusion

Although exertional rhabdomyolysis is typically caused by large-weight workouts, the disease can also be caused by low-weight workouts, as demonstrated by this case. It is the intensity of the workout, not the amount of weight used, that can cause rhabdomyolysis. Physicians must be aware of this when they examine patients complaining of muscle pain and weakness associated with rhabdomyolysis. The primary cause of the disease is muscle death, which can be caused in a myriad of ways.
